# Vaginal Polyelectrolyte Layer-by-Layer Films Based on Chitosan Derivatives and Eudragit^®^ S100 for pH Responsive Release of Tenofovir

**DOI:** 10.3390/md18010044

**Published:** 2020-01-09

**Authors:** Raúl Cazorla-Luna, Araceli Martín-Illana, Fernando Notario-Pérez, Luis Miguel Bedoya, Aitana Tamayo, Roberto Ruiz-Caro, Juan Rubio, María-Dolores Veiga

**Affiliations:** 1 Department of Pharmaceutics and Food Technology, Faculty of Pharmacy, Complutense University of Madrid, 28040 Madrid, Spain; racazorl@ucm.es (R.C.-L.); aracelimartin@ucm.es (A.M.-I.); fnotar01@ucm.es (F.N.-P.); rruizcar@ucm.es (R.R.-C.); 2 Department of Pharmacology, Pharmacognosy and Botany, Faculty of Pharmacy, Complutense University of Madrid, 28040 Madrid, Spain; lmbedoya@ucm.es; 3 Institute of Ceramics and Glass, Spanish National Research Council, 28049 Madrid, Spain; aitanath@icv.csic.es (A.T.); jrubio@icv.csic.es (J.R.)

**Keywords:** chitosan lactate, chitosan tartrate, chitosan citrate, Eudragit^®^ S100, layer-by-layer film, mucoadhesive film, Tenofovir controlled release, pH responsive release, vaginal preexposure prophylaxis, HIV sexual transmission

## Abstract

Women are still at high risk of contracting the human immunodeficiency virus (HIV) virus due to the lack of protection methods under their control, especially in sub-Saharan countries. Polyelectrolyte multilayer smart vaginal films based on chitosan derivatives (chitosan lactate, chitosan tartate, and chitosan citrate) and Eudragit^®^ S100 were developed for the pH-sensitive release of Tenofovir. Films were characterized through texture analysis and scanning electron microscopy (SEM). Swelling and drug release studies were carried out in simulated vaginal fluid and a mixture of simulated vaginal and seminal fluids. Ex vivo mucoadhesion was evaluated in bovine vaginal mucosa. SEM micrographs revealed the formation of multilayer films. According to texture analysis, chitosan citrate was the most flexible compared to chitosan tartrate and lactate. The swelling studies showed a moderate water uptake (<300% in all cases), leading to the sustained release of Tenofovir in simulated vaginal fluid (up to 120 h), which was accelerated in the simulated fluid mixture (4–6 h). The films had high mucoadhesion in bovine vaginal mucosa. The multilayer films formed by a mixture of chitosan citrate and Eudragit^®^ S100 proved to be the most promising, with zero toxicity, excellent mechanical properties, moderate swelling (<100%), high mucoadhesion capacity, and Tenofovir release of 120 h and 4 h in vaginal fluid and the simulated fluid mixture respectively.

## 1. Introduction

Acquired immunodeficiency syndrome (AIDS) is still the leading cause of death among young women. According to United Nations Joint Programme on HIV/AIDS (UNAIDS), 460 adolescent women contract human immunodeficiency virus (HIV) each day and 350 women of the same age group die weekly of AIDS-related complications. In fact, almost 80% of people infected with HIV in the 10–19 age group in sub-Saharan Africa in 2017 were women [1]. Unfortunately, women in these countries do not benefit from options to prevent the sexual transmission of HIV such as condoms, due to gender differences which prevent them from negotiating with their sexual partners. This highlights the need to develop prevention systems that women can initiate without their partner’s consent [2]. In this scenario, topical preexposure prophylaxis (PrEP) with antiretroviral drugs is one option to prevent the sexual transmission of HIV, since they can be applied in the vagina and serve as a method of protection that can be controlled by women themselves, thus empowering them in the fight against HIV-1 infection without the consent of their sexual partner [3].

Tenofovir (TFV) is an ideal candidate for use as a topical microbicide for preventing the sexual transmission of HIV due to its efficacy, long half-life, and safety profile [4]. Clinical trials have been carried out with this drug in different dosage forms such as rings [5] and gels [6].

However, adherence is central to PrEP effectiveness, and it is essential to develop products that are easy to use and support high adherence [7]. Vaginal films are emerging as a promising option among the pharmaceutical dosage forms in developmental stages, as they are preferred over other dosage forms due to their advantages of portability, storage and handling, and also ensure comfort and ease of insertion [8,9]. Fast dissolving films based on TFV have been produced and show lower leakage and similar vaginal drug concentrations to those obtained with vaginal gels for pericoital administration. Nevertheless, sustained release formulations must be developed to provide lasting protection to women [7].

One approach to obtaining films for the sustained release of drugs is the use of the layer-by-layer (LbL) technique, which produces films composed of two or more layers of different polymers. This offers great versatility, since it combines the properties of the constituent polymers of each layer [10]. For instance, the combination of a highly mucoadhesive polymer to increase vaginal retention [11] with another polymer capable of modulating the release of the drug [12] can achieve effective and long-lasting drug release in the vaginal mucosa. The combination of polyanionic polymeric layers with polycationic polymeric layers by means of this technique also produces polyelectrolyte multilayers (PEM) [13,14], with the advantage that these polymers have a pH-dependent behaviour [15,16]. This is of great interest when developing formulations for the prevention of sexual transmission of HIV, since accelerating vaginal drug release at the time of ejaculation can increase the effectiveness of the protection [17].

Among the polycationic polymers that are being explored, chitosan is a copolymer composed of β(1→4)-linked 2-acetamido-2-deoxy-β-d-glucopyranose (N-acetylglucosamine) and α(1→4)-linked 2-amino-2-deoxy-β-d-glucospyranose (glucosamine). Chitosan has been widely explored in the development of several pharmaceutical dosage forms such as tablets [11,18], hydrogels [19] and bigels [17], and attempts have recently been made to develop chitosan-based films as drug delivery systems [20,21]. Chitosan has mucoadhesive properties and antimicrobial activity; is from a renewable source and is completely devoid of toxicity, and has many applications in the pharmaceutical industry such as wound dressing and drug delivery systems [22,23]. This polymer has a pH-dependent solubility due to the presence of the amine groups in its structure [24], and the protonation of these groups in dilute acids allows the gelation of the polymer. Acetic acid is widely used among the various acids applied for the gelation of chitosan [25], although its strong and unpleasant smell induces rejection when applying the formulations [26]. For this reason, other acids are beginning to be used that allow the gelation of chitosan with better organoleptic properties, such as lactic acid [26], tartaric acid [27], and citric acid [28]. It has even been proven that the dilute acid used to dissolve the chitosan can condition the polymer’s properties [29].

In order to improve its properties, it possible to add suitable fillers [30,31] or combine it with different polymers such as hypromellose [32], sodium alginate [33], pectin [18], poly(vinyl alcohol) [34] and different types of Eudragit^®^ [35,36], among which it is particularly worth highlighting Eudragit^®^ S100 (ES100), a polyanionic copolymer derived from metacrylic acid and methyl metacrylate. It is non-soluble in acids and water but soluble in dissolutions with a pH of over 7 [37]. This behaviour makes it an interesting candidate for the development of pH-sensitive drug delivery systems for oral, ocular, vaginal and topical administration [38]. Mixtures of chitosan and ES100 have been explored with promising results, due to their ability to form polyelectrolyte complexes [39].

Although nowadays films are a promising tool for vaginal administration, the development of controlled release films is a field yet to be explored. Furthermore, the use of pH-sensitive polymers may allow obtaining novel drug delivery systems for the prevention of sexually transmitted diseases [40]. Against this backdrop, the aim of this study is to develop PEM vaginal films based on optimized chitosan derivatives and ES100 using the LbL technique and evaluate them through scanning electron microscopy, texture analysis, swelling, ex vivo mucoadhesion and drug release tests and materials citotoxicity. These films would allow a high mucoadhesion capacity due to the presence of the chitosan-based layer, and a sustained release in the vaginal environment thanks to the ES100 layer. After ejaculation during intercourse, the release of the drug would accelerate, thus maximizing the effectiveness of the formulations in the prevention of the sexual transmission of HIV.

## 2. Results and Discussion

### 2.1. Characterization of Chitosan Gels

**Figure 1:**
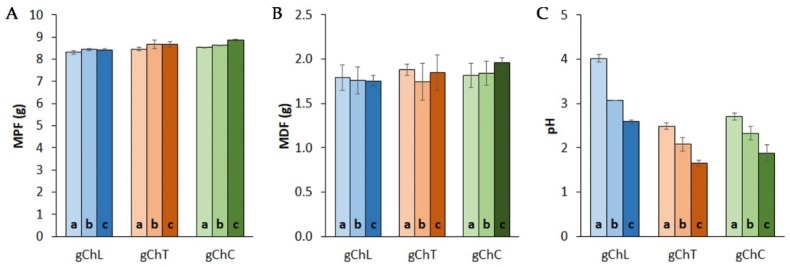
Results of the characterization of the chitosan gels prepared with lactic acid (gChL), tartaric acid (gChT) and citric acid (gChC) at different concentrations (0.25 M (a), 0.5 M (b) and 1 M (c)). (**A**) and (**B**) show the texture analysis results (maximum penetration force (MPF) and maximum detachment force (MDF) respectively). (**C**) shows the pH values obtained for the gels.

The results of the characterization of the gels are shown in Figure 1.

According to these data, all the gels had identical results for maximum penetration force (MPF, Figure 1A) and maximum detachment force (MDF, Figure 1B), indicating that the consistency of chitosan gels, represented by the MPF, is not modified by the acid used for its gelation. The adhesiveness of the gel, represented by the MDF, is also equal in all the gels. These properties are therefore characteristic of the polymer and are not modified by the acid. Previously published results show that pure water has similar values of MPF and MDF (7.90 ± 00 g and 1.49 ± 0.07 g) [41], so at these concentrations, chitosan gels have a similar consistency and adhesiveness to pure water. However, a comparison of the pH of the different gels in Figure 1C reveals some differences. In all cases an increase in the acid concentration leads to a decrease in the pH of the gel. The gels prepared with lactic acid show the highest values of pH (≈2.5–4), while citric acid and tartaric acid generate more acid gels, with pH values in the range of ≈2–3. According to the literature, the use of acidic substances in the vaginal environment may be beneficial for the prevention of sexually transmitted infections [42,43]. These formulations would therefore allow the acidification of the medium after administration, making them interesting candidates for the development of formulations for the prevention of sexual transmission of HIV.

### 2.2. Chitosan Derivative-Based Films

#### 2.2.1. Attenuated Total Reflection Fourier Transform Infrared (FTIR-ATR) Spectroscopy

**Figure 2:**
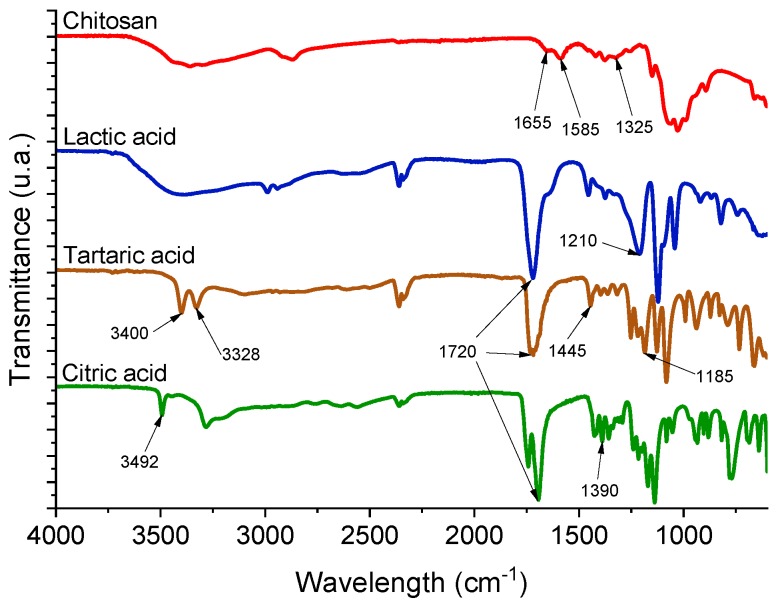
Spectra obtained through FTIR-ATR for the raw materials used to manufacture the chitosan derivative films.

Figure 2 shows the spectra obtained for the raw materials. In the spectrum corresponding to raw chitosan, bands are observed at 1655 cm^−1^ and 1325 cm^−1^, corresponding to the C=O bond of amide I and the N-H bond of amide III respectively. A band appears at 1585 cm^−1^, corresponding to the free amine [44]. In the lactic acid spectrum, a peak can be observed at 1720 cm^−1^ corresponding to the C=O bond of the acid group, and a band at 1210 cm^−1^ that can be attributed to the C-O carboxylic acid bond [45]. In tartaric acid, the peak observed at 1720 cm^−1^ corresponds to the C=O bond of the carboxylic acid. Another peak at 1445 cm^−1^ corresponds to the O-H bond of the carboxylic groups. The peaks at 1185 cm^−1^, 3328 cm^−1^ and 3400 cm^−1^ are also attributable to O-H links [46]. In the citric acid spectrum, the peaks that appear around 1720 cm^−1^ are due to the C=O bonds of carboxylic acids. A band corresponding to the O-H bond of carboxylic acids is observed at 1390 cm^−1^. The 3492 cm^−1^ peak is also attributable to free hydroxyls [47].

**Figure 3:**
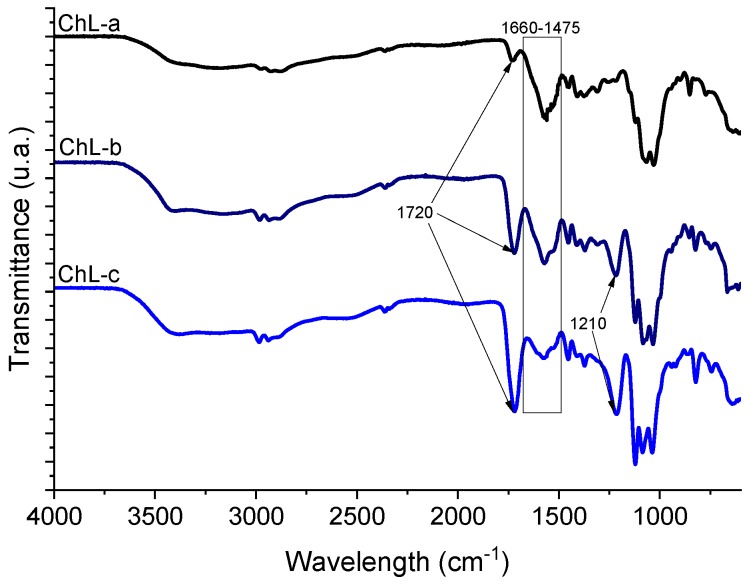
Spectra obtained through FTIR for the films manufactured by solvent casting chitosan gels prepared with different concentrations of lactic acid: 0.25 M (ChL-a), 0.5 M (ChL-b), and 1 M (ChL-c).

**Scheme 1:**
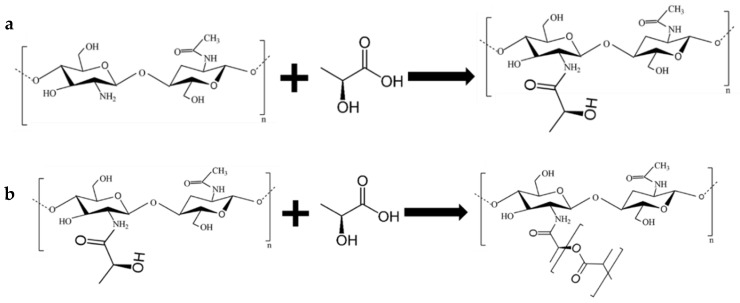
Chemical reaction between chitosan and lactic acid. First, the carboxylic group of lactic acid reacts with the amine group of chitosan, forming the corresponding amide (henceforth chitosan lactate) (**a**). If there is an excess of lactic acid, the carboxylic groups of free molecules react with the free hydroxyl of the lactic group in chitosan lactate, forming polylactate groups (**b**).

The spectra corresponding to chitosan films prepared with different amounts of lactic acid (ChL-a, ChL-b and ChL-c) are shown in Figure 3. All have a broad band between 1660–1475 cm^−1^, caused by the overlapping of the chitosan amide (1655 cm^−1^) and the C=O bond corresponding to the amide formed with lactic acid [48]. Since the carbonyl in the acid (1720 cm^−1^) changes to amide, a reduction in the intensity of this peak can be seen in samples ChL-a and ChL-b. The peak is also observed to decrease to 1210 cm^−1^, due to the replacement of hydroxyl in the formation of this amide (Scheme 1a). When the amount of lactic acid in the film is increased (especially in ChL-c), esters form between the lactic acid molecules, creating oligomers of polylactic acid (Scheme 1b) [48] and causing the intensity of the band to increase at 1720 cm^−1^ due to the C=O bonds corresponding to the esters. The formation of these esters is favoured by the high concentration of lactic acid, so this peak is more intense than the peak attributable to the C=O of the amide. The band at 1210 cm^−1^ also increases, indicating the presence of the C-O bond, which is absent in the amide but present in the ester [48].

**Figure 4:**
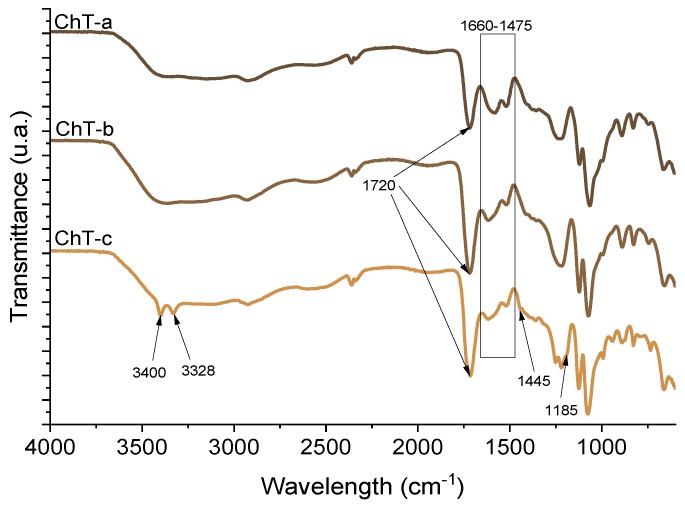
Spectra obtained through FTIR for the films manufactured by solvent casting chitosan gels prepared with different concentrations of tartaric acid: 0.25 M (ChT-a), 0.5 M (ChT-b) and 1 M (ChT-c).

**Scheme 2:**
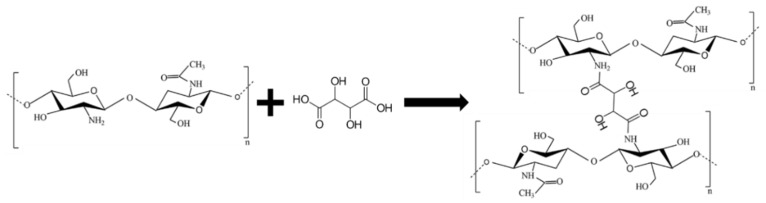
Chemical reaction between chitosan and tartaric acid. The carboxylic groups of tartaric acid react with the amine group of chitosan, forming the corresponding amide (henceforth chitosan tartrate). The complete reaction with the two carboxylic groups in the molecule of tartaric acid leads to the crosslinking of chitosan.

The spectra corresponding to chitosan films prepared with tartaric acid are shown in Figure 4. A broad band appears between 1660 cm^−1^ and 1475 cm^−1^, with two peaks attributable to the amide group [49]. In ChT-a and ChT-b, the peak disappears at 1445 cm^−1^, corresponding to the O-H bond of the carboxylic acid of the tartaric acid. Other peaks that disappear with the lowest proportions of tartaric acid are 1185 cm^−1^, 3400 cm^−1^ and 3328 cm^−1^, which also indicates the replacement of hydroxyls in tartaric acid and confirms the formation of the amide between chitosan and tartaric acid (Scheme 2) [27]. The absence of these peaks corresponding to the O-H bonds of the carboxylic acids indicates that all acid groups react with the chitosan amines, suggesting that tartaric acid acts as a crosslinker of the chitosan chains. The peaks corresponding to O-H bonds (1445 cm^−1^, 1185 cm^−1^, 3328 cm^−1^, and 3400 cm^−1^) [46] reappear with the highest amount of tartaric acid (ChT-c), indicating that not all the tartaric acid is used in the reaction at this proportion.

**Figure 5:**
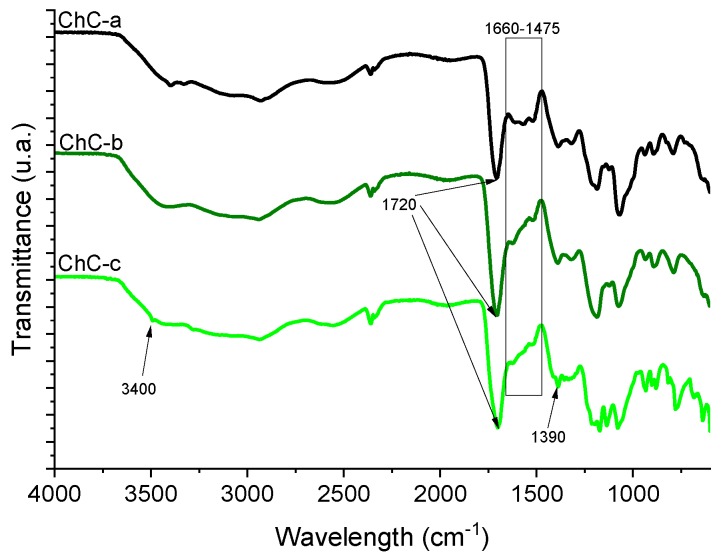
Spectra obtained through FTIR for the films manufactured by solvent casting chitosan gels prepared with different concentrations of citric acid: 0.25 M (ChC-a), 0.5 M (ChC-b), and 1 M (ChC-c).

**Scheme 3:**
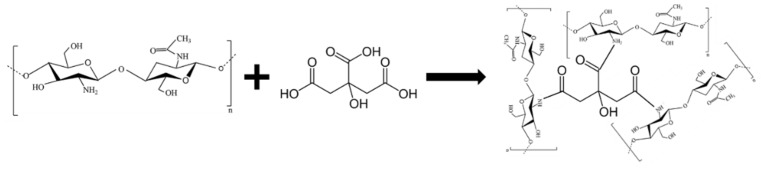
Chemical reaction between chitosan and citric acid. The carboxylic groups of citric acid react with the amine group of chitosan, forming the corresponding amide (henceforth chitosan citrate). The complete reaction with the three carboxylic groups in the citric acid molecule leads to the crosslinking of chitosan.

According to the spectra obtained for the chitosan films prepared with citric acid shown in Figure 5, a wide band appears between 1660 cm^−1^ and 1475 cm^−1^, attributable to the formation of the amide group [50]. The bands at 1390 cm^−1^ and 3492 cm^−1^ disappear in samples ChC-a and ChC-b due to the replacement of all free carboxylic hydroxyls by the chitosan amines. This indicates that amide is formed between chitosan and citric acid, and that citric acid acts as a crosslinker for chitosan chains through an amide formed between both compounds (Scheme 3) [51]. The peaks at 1390 cm^−1^ and 3492 cm^−1^ reappear in the sample with the highest proportion of citric acid (ChC-c), suggesting that the reaction with citric acid in these proportions is incomplete.

#### 2.2.2. Appearance and Mechanical Properties

The films made with chitosan derivatives were visually evaluated. The pliability and organoleptic characteristics of each batch are shown in Table 1.

The mechanical properties of the system must be taken into account when developing films for vaginal administration, as they can determine comfort and acceptability for the patient and may even be related to the performance of the film once administered. In the case of ChL-c, the formation of polylactic acid is so pronounced that the film is sticky and becomes impossible to handle. ChT-c and ChC-c films were also observed to be rigid and irregular, which can be attributed to the excess of acid during the reaction, as corroborated by FTIR studies (Section 2.2.1). The excess of tartaric acid or citric acid stiffens the film, as they are solid substances. These films were therefore discarded from further studies.

The comparison of the other films in terms of the acid used for their preparation revealed that the films with chitosan lactate had the lowest deformation capacity, while those containing chitosan tartrate or chitosan citrate had a higher capacity. This may be related to the ability of tartaric acid and citric acid to act as crosslinkers, possibly facilitating the mobility of the chitosan chains [52]. In all cases the films prepared with the diluted acids at 0.5 M (CaL-b, ChT-b, and ChC-b) were more flexible than those obtained with the diluted acids at 0.25 M (ChL-a, ChT-a, and ChC-a).

#### 2.2.3. Drug Release Assessment

**Figure 6:**
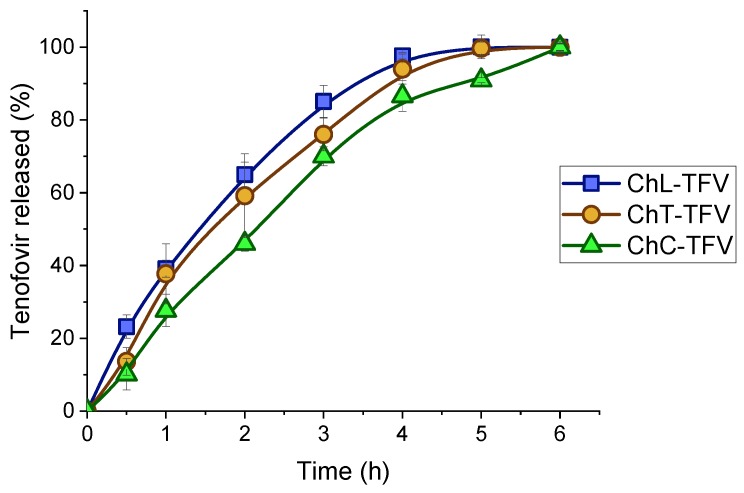
Tenofovir release from the films containing chitosan lactate (ChL-TFV), chitosan tartrate (ChT-TFV) and chitosan citrate (ChC-TFV).

Chitosan derivative films prepared with the previously determined optimal concentration of diluted acids (ChL-b, ChT-b, ChC-b) were loaded with 30 mg of TFV (batches ChL-TFV, ChT-TFV and ChC-TFV). The drug release profiles from these chitosan derivative-based films are shown in Figure 6.

In all cases, the entire Tenofovir dose is released in 6 h, revealing the inability of these films to control the release of the drug. The crosslinking of chitosan chains generates complex three-dimensional structures, so ChC-TFV and ChT-TFV could be expected to show a greater capacity for controlled drug release than ChL-TFV, which should release the drug at the fastest rate. While this behaviour is indeed observed, there are insufficient differences between the formulations. This can be explained by the fact that they form gels with a very low consistency, as was observed in the gel characterization studies (Section 2.1). For this reason, and to modulate the gelation rate of chitosan derivatives, and consequently the release of TFV, it was decided to prepare LbL films using ES100.

### 2.3. Layer-by-Layer Films

**Figure 7:**
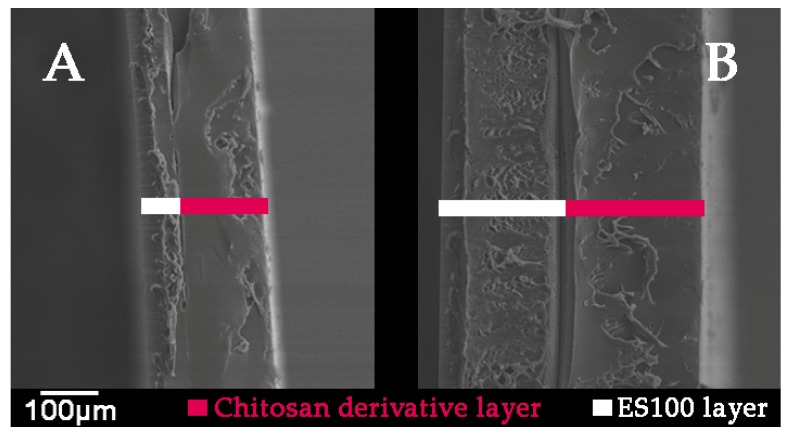
Micrographs of the cross-section of LbL films at 100 times magnification, with 75 mg of ES100 (**A**) and 150 mg of ES100 (**B**).

In order to improve the properties of the chitosan derivatives, LbL films were prepared using the previously selected chitosan derivative-based films (ChL-TFV, ChT-TFV and ChC-TFV). Six different batches were obtained by adding a layer of ES100 (plasticized with Triethylcitrate (TEC)) of 75 mg (ChL/E-a, ChT/E-a and ChC/E-a) or 150 mg (ChL/E-b, ChT/E-b and ChC/E-b). As seen in the SEM micrographs (Figure 7), the layers were tightly joined and LbL films were obtained.

#### 2.3.1. Texture Analysis

**Figure 8:**
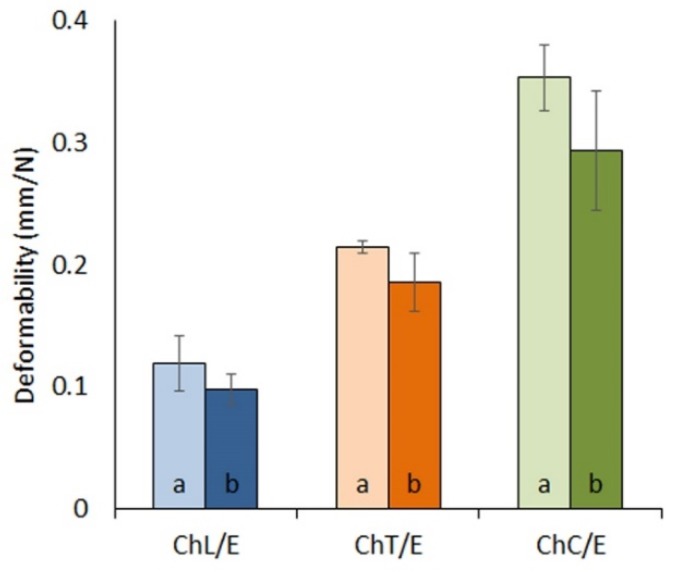
Results of the deformability of the LbL films containing chitosan derivatives and ES100, determined through texture analysis.

Figure 8 shows the deformability of the films, determined through texture analysis.

The films containing chitosan lactate (ChL/E 1 and ChL/E 2) have the lowest deformability values. Films based on chitosan tartrate (ChT/E 1 and ChT/E 2) exhibit a higher deformation capacity compared to ChL/E films. Chitosan citrate-based films (ChC/E 1 and ChC/E 2) have the highest deformability. According to the ANOVA processing, the differences are statistically significant (*p*-value = 1.85 × 10^−11^). The proportion of ES100 also conditions the deformability of the systems with statistically significant differences (*p*-value = 7.73 × 10^−3^), which can be attributed to the thickness of the ES100 layer. It is therefore necessary to apply a slightly greater force to obtain the same deformation when this layer is double the thickness.

These results can be explained by the fact that lactic acid is unable to crosslink chitosan chains. Conversely, tartaric acid and citric acid act as crosslinkers for chitosan, which is why these films (ChT/E and ChC/E) present better mechanical properties, as the mobility of the polymer chains may be enhanced by the presence of crosslinkers [52]. Among the films evaluated, those based on chitosan citrate (ChC/E-a and ChC/E-b) can therefore be considered to have the best mechanical properties, followed by films based on chitosan tartrate (ChT/E-a and ChT/E-b); films prepared with chitosan lactate (ChL/E-a and ChL/E-b) are the least appealing. However, all the films show good properties for handling and application, since they are highly resistant and flexible.

#### 2.3.2. Swelling Behaviour

**Figure 9:**
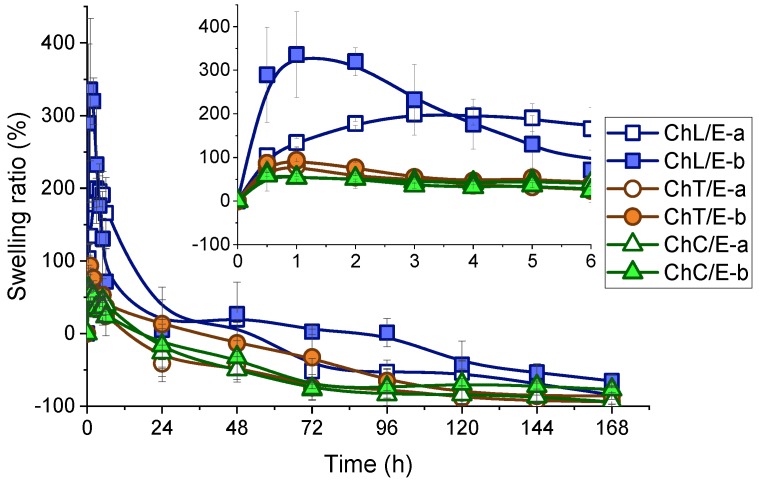
Swelling profiles of the LbL films in SVF.

The results of the swelling behaviour assessment in simulated vaginal fluid (SVF) are shown in Figure 9. In the case of films based on chitosan lactate and ES100, it can be observed that the maximum amount of water imbibed is significantly greater when the ES100 layer is thicker. ChL/E-a shows a maximum swelling ratio (SR_max_) of ≈ 200% while ChL/E-b has a SR_max_ of ≈ 320%. The complete dissolution of the derivative (which leads to the constant weight of the formulation) occurs in both cases at 168 h. These results show a certain incompatibility between the layers, since an increase in the thickness of the ES100 layer leads to an increase in the water uptake rate and in the SR_max_.

The profiles of the batches based on chitosan tartrate (ChT/E-a, ChT/E-b) are practically overlapping, which implies that the presence of the ES100 layer does not condition the swelling process in this medium. Their low SR_max_ (less than 100%) indicate that these films swell very little. The swelling profiles of the films based on chitosan citrate and ES100 (ChC/E-a and ChC/E-b) also overlap, suggesting that the thickness of the ES100 layer does not condition the water uptake capacity, as observed in ChT/E films. These films also have the lowest SRmax values in SVF (less than 75%).

These results can be explained by the fact that that the three-dimensional structure generated by crosslinkers in chitosan hinders water penetration [53], so although CL/E films show a higher swelling capacity, all the films could be useful for developing mucoadhesive vaginal formulations, since they swell so little that they would be extremely comfortable for the patient.

#### 2.3.3. Ex Vivo Mucoadhesion

**Figure 10:**
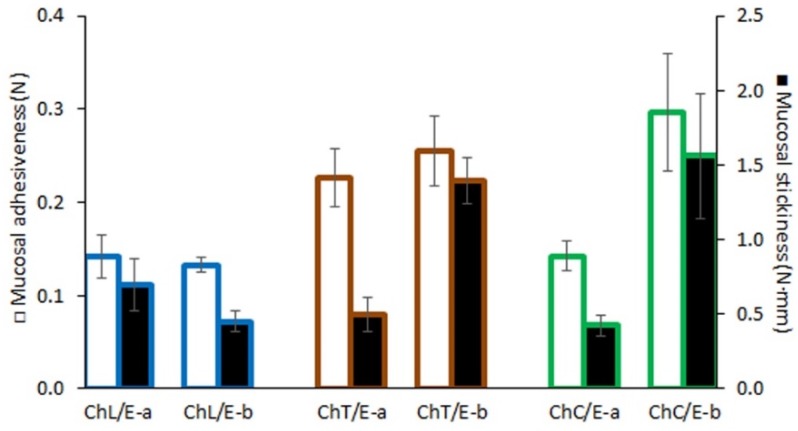
Ex vivo mucosal adhesiveness and stickiness obtained for the LbL films, determined in excised vaginal bovine mucosa and SVF. Mucosal adhesiveness is represented by the white bars (values on the left axis), and mucosal stickiness is by the black bars (values on the right axis).

Ex vivo mucoadhesion was determined through texture analysis on excised bovine vaginal mucosa in SVF, and the data are shown in Figure 10. According to the results, ChL/E films have the lowest values for mucosal stickiness (represented by the work required to detach them) and adhesiveness (detachment force). This is because these films show the highest SR_max_. According to previously published results [54], the amount of water imbibed is inversely related to the mucoadhesion capacity of a formulation, as water hinders the interaction between the polymer and the mucosa. ChT/E and ChC/E therefore show higher values for mucosal stickiness, adhesiveness or both. The results reveal that the amount of ES100 also conditions the mucoadhesion capacity of the ChT/E and ChC/E films, probably because ES100 acts as a support capable of holding the chitosan derivative. Thus the greater the amount of ES100, the greater the ability of the chitosan to bind to the mucosa. In the case of ChC/E films, the difference between the formulations points to a synergy between the layers, and shows that increasing the thickness of the ES100 layer substantially improves the mucoadhesion capacity of the chitosan derivative-based layer. Finally, it should be noted that ChC/E-b shows a slightly higher mucoadhesion capacity than ChT/E-b. The ChC/E-b film thus has a high mucoadhesion capacity, superior to the formulations previously developed, which remained mucoadhered for 120 h [18].

#### 2.3.4. Drug Release

**Figure 11:**
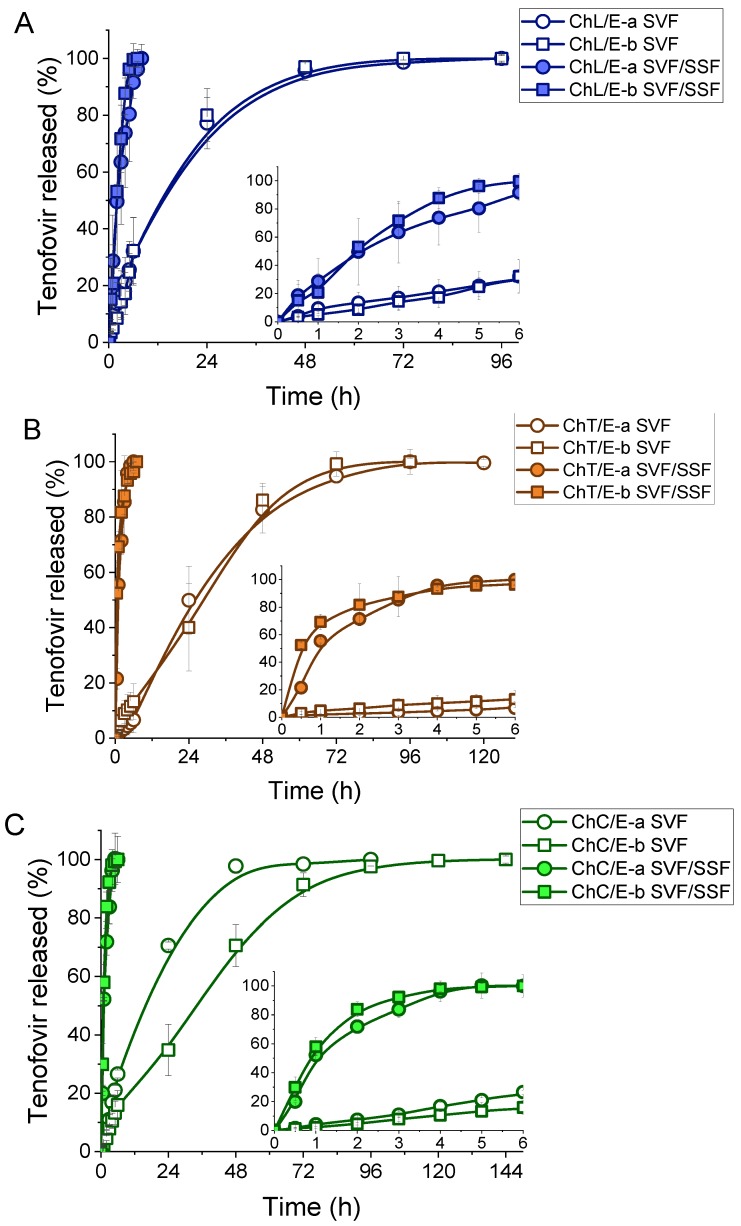
TFV release profiles in SVF and SVF/SSF from the LbL films based on chitosan lactate (**A**), chitosan tartrate (**B**) and chitosan citrate (**C**). The first 6 h of testing are amplified in the lower right corner of each graph.

Drug release data from the systems in SVF and SVF/SSF are shown in Figure 11. All the systems exhibited an ability to release the drug in a controlled manner in SVF (in all cases the release lasts more than 48 h), while a burst release is observed in SVF/SSF for all the systems (the complete amount of drug is released in less than 6 h).

According to Figure 11A, films based on chitosan lactate (ChL/E-a and ChL/E-b) show a sustained release of TFV of up to 48 h in SVF and 6h in SVF/SSF. In films based on chitosan tartrate (ChT/E-a and ChT/E-b), whose release profiles are shown in Figure 11B, TFV release in SVF extends up to 96 h, and 4 h in SVF/SSF. In films containing chitosan citrate (ChC/E-a and ChC/E-b), the release of TFV in SVF is maintained for 120 h, while in SVF/SSF the totality of the drug is released in 4 h, as seen in Figure 11C. These findings are closely related to the swelling behaviour of LbL films, where the swelling of ChL/E-a and ChL/E-b is greater in SVF; as they have the highest penetration of water in the system, the TFV molecules are more easily accessed by the water in the medium, accelerating the drug dissolution. The SRmax is slightly higher in ChT/E films than in ChC/E films, which explains why ChC/E films show the most extended release and also confirms that crosslinking chitosan improves the capacity to control drug release, as chitosan tartrate and chitosan citrate-based films show better controlled release than chitosan lactate-based films. A comparison of tartaric acid and citric acid suggests that citric acid is the best crosslinker and has the most persistent TFV release. However, this confirms the need for an upper layer based on ES100 to enhance the properties of the crosslinked chitosan.

According to the f_2_ statistic (Table 2), no significant differences are observed in the release profiles for ChL/E and ChT/E in SVF when comparing the release from films in SVF based on the amount of ES100 in the upper layer. As seen in the swelling test results, this implies that the entry of water into the formulation is modulated by the presence of ES100 regardless of the thickness of the layer, which is key to the capacity to modulate drug release. However, ChC/E films show differences in release depending on the thickness of the ES100 layer, once again indicating a synergy between the chitosan citrate and ES100, as was observed in the mucoadhesion studies (Section 2.3.3). In SVF/SSF the entry of water is so accelerated that the difference in release based on the thickness of this layer is negligible, and they all release the drug in similar times. It is verified by statistical f_2_ that the release profiles of LbL films are strongly conditioned by pH, due to the fact that ES100 is a pH-sensitive polymer that becomes soluble in aqueous media at a pH of over 7 [55]. In consequence, the dissolution of the ES100 in the medium leaves the chitosan solely responsible for the release of Tenofovir, and this polymer will release the drug in less than 8 h, as already verified in the release profiles of chitosan derivative-based films (Section 2.2.3).

The interest of this finding lies in the ability of the ChC/E-b film to offer women effective protection for a maximum of 120 h, generating a much higher concentration of TFV in the vaginal environment after ejaculation during sexual intercourse. This points to a high efficiency in the prevention of sexual transmission of HIV, and therefore justifies future evaluations.

### 2.4. Material Cytotoxicity

**Figure 12:**
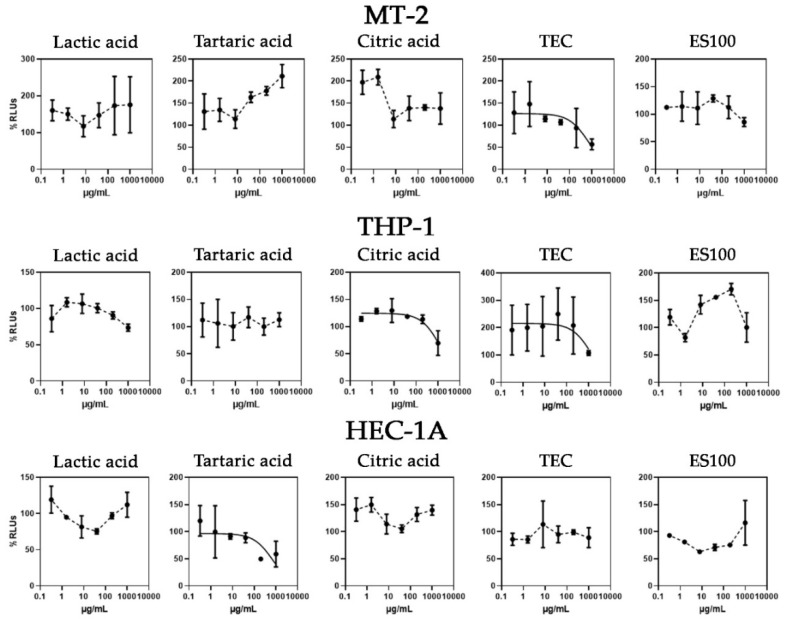
Graphic representation of the cytotoxic evaluation of lactic acid, tartaric acid, citric acid, TEC and ES100 in MT-2, THP-1 and HEC-1A cells. Cell viability is expressed as a percentage of living cells (%RLUs) as compared to a non-treated control (100%). RLUs: relative luminescence units.

To study cell toxicity, the materials were incubated in culture media at 37 °C and 5% CO_2_ for 48 h before the assay to ensure that any potential toxic component would be present in the dilutions to be tested. The cell culture was then treated with a suspension of base 5 serial dilutions of the most concentrated suspension (1000 µg/mL). Experiments were performed in lymphoblastic (MT-2) and macrophage-monocyte (THP-1) derived cell lines to evaluate toxicity on the immune cells present in vaginal or uterine mucosae, and in a uterine epithelial cell line (HEC-1A) to evaluate the potential damage to mucosal integrity (Figure 12).

As shown in Figure 12 and Table 3, lactic acid and ES100 were biocompatible in the three cell types, displaying CC_50_ values of over 1000 µg/mL, the maximum concentration tested. Citric acid CC_50_ value in THP-1 was around 1000 µg/mL, which is high enough to rule out a toxic effect in vivo, since it must pass through the epithelium layers at that concentration to reach the monocytes/macrophages that are only present on the inside of the epithelium. The same can be said of TEC since its CC_50_ of around 1000 µg/mL is only seen in MT-2 cells, which is a lymphocyte type cell line. The only compound showing cell toxicity below 1000 µg/mL was tartaric acid, with a CC_50_ of around 200 µg/mL in HEC-1A cells, with no effect on THP-1 and MT-2 cells. In this case tartaric acid is toxic to the epithelium cells, which could lead to the disruption of the layer and to a potential inflammatory response, facilitating viral entry through the vaginal mucosa, as other substances as nonoxynol-9 have been found to do [56]. Therefore, although the concentration needed to obtain the toxic effect is quite high (200 µg/mL), tartaric acid should be avoided in any formulation to be applied to the vaginal and/or uterine epithelium.

## 3. Materials and Methods

### 3.1. Materials

Tenofovir (TFV, lot: FT104801401, MW: 287.21 g/mol, purity ≥ 98%) was provided by Carbosynth Limited (Berkshire, UK). Low molecular weight chitosan [18] (CH, lot: 0055790, MW ≈ 32KDa, purity ≥ 90%) was supplied by Guinama (Valencia, Spain). L(+)-Lactic acid (lot: 0001552193, MW: 90.08 g/mol, purity = 90.8%) was purchased from PanReac AppliChem (Barcelona, Spain). L(+)-Tartaric acid (lot: BCBW8050, MW: 150.09 g/mol, purity = 99.97%), citric acid (lot: BCBV9045, MW: 192.12 g/mol, purity ≥ 99.5%) and triethylcitrate (TEC, Lot: BCBN8745V, MW: 276.28 g/mol, purity = 99.9%) were supplied by Sigma Aldrich (Saint Louis, MO, USA). Eudragit^®^ S100 (ES100, lot: B071005090, MW = 135KDa [57]) was a kind gift from Evonik (Darmstadt, Germany). All other reagents used in this study were of analytical grade and used without further purification. Demineralized water was used in all cases.

### 3.2. Methods

#### 3.2.1. Manufacture and Characterization of Chitosan Gels

Diluted acids must be used to obtain chitosan gels in aqueous media [54]. 50 mL of chitosan gels were therefore prepared with three different organic acids (lactic acid, tartaric acid and citric acid) at three concentrations, as shown in Table 4. The gels obtained were stored for 24 h at room temperature to ensure the correct hydration of the polymer, then immediately evaluated.

The influence of the acid used and its concentration on the chitosan gels was studied by evaluating their textural properties with a TA.XTplus Texture Analyser (Stable Micro Systems, Surrey, UK) using a 5 kg load cell, and following a previously described method [54]. A 20 mm-diameter stainless-steel probe with an activation force of 2 g was introduced in each gel at a rate of 0.5 mm/s to a depth of 15 mm, and returned to the initial height at the same rate. 500 points per second were monitored during data collection, and the maximum penetration force (MPF)—an accurate prediction of gel consistency—was calculated. The maximum detachment force (MDF), said to be a predictor of adhesive performance, was also recorded. The test was performed in triplicate for all the gels evaluated.

The pH of the gels was also determined using a pH-meter (GLP 21, Crison Instruments^®^ S.A., Barcelona, Spain).

#### 3.2.2. Chitosan Derivative-Based Films

##### Film Manufacture

Films were obtained from 5 mL of the previously prepared and evaluated gels using the solvent casting method [9] (Table 5) in silicon moulds. Once the solvent was completely dried at room temperature, 45-mm diameter circular films were stored until further analysis.

The films were visually assessed to determine the optimum concentration of the different acids to obtain films based on chitosan derivatives. Their organoleptic properties were observed and their pliability was determined by folding the films, considering their capacity to deform and recover their form. The optimal proportions were then prepared using the same method with the addition of 30 mg of TFV to the gel. The amount of TFV for the development of the films was carefully selected according to literature and previous evaluations. Among the different dosage forms for the vaginal administration of this drug in clinical trials, lower and higher doses have been tested, showing adequate efficacy and security [7]. It has also been proved that extremely low cervicovaginal fluid concentration of TFV (1000 ng/mL) confer protection against HIV in women, ensuring the efficacy of these films during the release period [58]. As for the security of the films, it was confirmed in cytotoxicity studies that the dose included is not toxic for the vaginal tissue [59].

##### Attenuated Total Reflection Fourier Transform Infrared (FTIR-ATR) Spectroscopy

Attenuated total reflection Fourier transform infrared (FTIR-ATR) spectroscopy was used to characterize raw materials and chitosan derivative-based films with a Perkin-Elmer spectrophotometer, equipped with a MIRacle™ accessory designed for measurements (Perkin-Elmer). 16 scans were recorded for all the spectra at a resolution of 4 cm^−1^.

##### Drug Release

Drug release was evaluated following the method described by Sánchez-Sánchez et al. [60]. Each sample was inserted in a borosilicate glass bottle with a 45 mm diameter base containing 80 mL of SVF and then placed in a shaking water bath (37 °C, 15 opm). Samples were taken every hour until the release of TFV was completed. 5 mL aliquots were removed and filtered, and the medium was replaced with the same volume of SVF at the same temperature. TFV concentrations were quantified by UV spectroscopy at a wavelength of 260 nm (Abs_SVF_ = 482.05·C(mg/mL)−0.0019; r^2^ = 0.9996) in an Evolution 60S spectrophotometer (Thermo Scientific, Kyoto, Japan). The test was performed in triplicate.

#### 3.2.3. Layer-by-Layer Films

##### Manufacture

Although the chitosan derivative-based films had an acceptable texture, they appear unable to sufficiently modulate the release of the drug. The optimal proportions of each chitosan derivative-based film were selected to prepare LbL films, adding different proportions of ES100 to prepare the second layer (Table 6). Since ES100 is an acidic polymer, the interaction between the surface of the chitosan film and the film based on ES100 occurs through electrostatic interactions, and both layers become tightly joined. These systems were obtained using the solvent casting method. First, chitosan derivative-based films containing TFV were prepared according to the method described above, then after the complete evaporation of water, a solution of ES100 in 10 mL acetone was added with TEC as a plasticizer. When this solvent was completely dry at room temperature, the resulting films were stored until further assessment.

##### SEM Microscopy

To verify the structure of the film and the arrangement of the layers, the cross-sections of the films were analysed by electron microscopy using a field emission scanning electron microscope (JEOL JSM-6335F, Tokyo, Japan) at an accelerating voltage of 20 V and a work distance of 15 mm.

##### Texture Analysis

Although the pliability of the chitosan derivative-based films has already been assessed, the incorporation of the ES100-based layer may lead to a significant modification in the mechanical properties of the system. The texture of the LbL films was therefore evaluated according to a previously set up methodology [61] using a TA.XTplus Texture Analyser (Stable Micro Systems, Surrey, UK) with a 30 kg load cell. Before starting the experiments, each film was fixed to a support rig. A 5mm-diameter spherical stainless probe with an activation force of 5 g applied increasing force to each film to maintain a moving rate of 0.5 mm/s. 500 points per second were monitored during data collection, and the force applied (N) and the distance travelled by the probe (mm) were registered at each point. The measurement ended when the film burst at the maximum registered force. The distance travelled by the probe at the burst of the films was also recorded. The deformability of the systems was then determined as the average distance travelled by the probe when applying a force of 1 N.

All assays were performed in quadruplicate, and the data were statistically processed using two-way ANOVA (*p*-value 0.05) with the nature of the chitosan derivative and the proportion of ES100 as factors.

##### Swelling Behaviour

Swelling studies were performed on the LbL films to characterize the swelling of the formulations in SVF as a function of time.

The swelling processes of the different batches in SVF were analysed following the method described by Mamani et al. [62]. Fragments of each formulation with a diameter of 3 cm were fixed with cyanoacrylate adhesive to stainless steel discs of the same size, with the chitosan derivative-based layer facing the disc and the ES100 layer facing outwards, thus reproducing the expected conditions after administration. They were then placed in beakers containing 100 mL of SVF and introduced in a shaking water bath (37 °C, 15 opm). At given times (every hour during the first six hours and once a day up to constant weight), the discs were extracted from the medium and weighed after removing excess liquid. Swelling ratio (SR) was calculated according to Equation (1):

SR (%) = [(F_s_ − F_d_)/(F_d_·SF)]·100, (1)

where F_s_ and F_d_ correspond to the swollen and dry film weights respectively, and SF represents the swellable fraction of the film. All the assays were performed in triplicate.

##### Ex Vivo Mucoadhesion

The mucoadhesion force and work were assessed ex vivo using the TA.XTplus Texture Analyser (Stable Micro Systems) to check whether the formulations show sufficient mucoadhesion capacity to adhere to the vaginal mucosa at the time of administration, using a modification of a previously described method [17]. A 2 × 2 cm fragment of excised bovine vaginal mucosa (obtained from a local slaughterhouse) was fixed to the bottom of a Petri dish and hydrated with 5 mL of SVF. The LbL film was fixed to a 1 cm-diameter cylindrical probe, with the chitosan derivative layer facing the vaginal mucosa, as expected at the time of administration. The preparation was moved at a speed of 1 mm/s until it came into contact with the vaginal mucosa, applying a contact force of 500 g for 30 s. The probe was then separated from the sample at a speed of 1 mm/s up to the starting height of the test. The maximum force required to separate the film from the vaginal mucosa (detachment force) was recorded as the mucosal adhesiveness. The area under the curve between the force-distance profiles (detachment work) was determined, which is considered to be the mucosal stickiness. Each batch was evaluated in triplicate.

##### Drug Release

To verify that the release of TFV from the films is pH dependent, the drug release from the LbL films was evaluated in SVF (pH = 4.2) and in a SVF and simulated seminal fluid mixture (SVF/SSF, ratio 1:4, pH = 7.5 [63]) to reproduce the conditions after ejaculation during intercourse. Each sample was inserted in a borosilicate glass bottle with a 45-mm diameter base containing 80 mL of medium, with the hydrophilic layer of the film in contact with the glass and the hydrophobic layer in contact with the medium, as it would be positioned in the vagina. The preparation was then placed in a shaking water bath (37 °C, 15 opm). Samples were taken every hour during the first six hours and every day after that at given times. 5mL aliquots were removed and filtered, and the medium was replaced with the same volume of either SVF or SVF/SSF at the same temperature. TFV concentrations were quantified by UV spectroscopy at a wavelength of 260nm (Abs_SVF/SSF_ = 482.23·C(mg/mL)−0.0145; r^2^ = 0.9990) in an Evolution 60S spectrophotometer (Thermo Scientific, Kyoto, Japan). The test was performed in triplicate.

The release profiles of the LbL films were compared using a f_2_ statistic [64] in order to determine whether there are significant differences between the two media, and whether the thickness of the ES100 layer determines the release of the drug.

#### 3.2.4. Material Cytotoxicity

Three human cell lines were used: a lymphoblastic cell line, MT-2 [65], a macrophage-monocyte derived cell line, THP-1 (ATCC^®^ TIB-202) and a uterine/endometrial epithelial cell line, HEC-1A (ATCC^®^ HTB-112™) (kindly provided by Maria Angeles Muñoz). All the cell lines were cultured in RPMI 1640 medium supplemented with 10 % (*v/v*) fetal bovine serum, 2 mM L-glutamine and 50 mg/mL streptomycin (all Whittaker M.A. Bio-Products, Walkerville, MD, USA) at 37 °C in a humidified atmosphere of 5 % CO_2_. To detach the HEC-1-A cells, the medium was removed and the flask was rinsed during 10 min with 1 to 2 mL of trypsin 0.25%—EDTA 0.03% solution. The medium was replaced every three days after cell centrifugation at 1500 rpm for 5 min.

Cell toxicity was measured using the CellTiter Glo kit (Promega, Madison, WI, USA). Cells were incubated in 96-well plates at a density of 10 × 10^5^ cells per well (MT-2 and THP-1) and 2 × 10^4^ (HEC-1A) in complete medium. To assess the cytotoxic effect, cells were exposed to fresh medium containing different concentrations of lactic acid, tartaric acid, citric acid, TEC and ES100 suspensions, or the same concentration of PBS 1× as control. Experiments were performed in triplicate and the culture was maintained at 37 °C and 5% CO_2_ humidified atmosphere for 48 h. A standard method was followed to suspend the materials in PBS 1× [66]. After incubation for 48 h, the medium was removed from cell cultures and 50 µL of CellTiter Glo reactive was added to each well on the plate. Relative luminescence units (RLUs) were measured in a luminometer (Sirius, Berthold Detection Systems). Cytotoxic concentration 50 (CC_50_) values were calculated using GraphPad Prism Software (non-linear regression, log inhibitor versus response). The results of the cytotoxic assay are shown as the average of at least three individual experiments.

## 4. Conclusions

The gelation of chitosan in different diluted acids allows the production of chitosan derivative-based films with different mechanical properties without the need to include plasticizers. It has been confirmed that the crosslinking of the chitosan chains with tartaric acid or citric acid generates films with improved mechanical properties. The combination of these films with a polymer with pH-sensitive solubility (Eudragit^®^ S100) produces formulations that exhibit a pH-dependant Tenofovir release, high mucoadhesion, and a moderate swelling profile, which would make them comfortable for the patient.

Among the films obtained through the layer-by-layer technique, those based on the combination of chitosan citrate and Eudragit^®^ S100 in a 1:1 ratio (ChC/E-b) allow a sustained release of Tenofovir for up to five days in simulated vaginal fluid and release all the drug in less than 4 h after sexual intercourse, with very moderate swelling and a high mucoadhesion capacity. The materials used were also non-toxic in the vaginal environment. These formulations are thus a future option for the prevention of the sexual transmission of HIV in women.

## Figures and Tables

**Table 1:** Characteristics observed by visual evaluation of films based on chitosan derivatives.

Batch	Pliability	Organoleptic Characteristics	Comments
ChL-a	✔	Homogeneous. Translucent, yellowish and shiny. Odourless. Soft touch.	Although somewhat flexible, it breaks if folded in half.
ChL-b	✔✔	Homogeneous. Translucent, yellowish and shiny. Odourless. Soft touch.	
ChL-c	✖	Homogeneous. Translucent, yellowish and shiny. Odourless. Sticky touch.	The film can barely be handled due to its stickiness.
ChT-a	✔✔	Homogeneous. Translucent, yellowish and shiny. Odourless. Soft touch.	
ChT-b	✔✔✔	Homogeneous. Translucent, yellowish and shiny. Odourless. Soft touch.	
ChT-c	✖	Heterogeneous. Opaque, yellowish and matt. Odourless. Rough touch.	Completely rigid, it breaks when even slight force is applied.
ChC-a	✔✔	Homogeneous. Translucent, yellowish and shiny. Odourless. Soft touch.	
ChC-b	✔✔✔	Homogeneous. Translucent, yellowish and shiny. Odourless. Soft touch.	
ChC-c	✖	Heterogeneous. Opaque, yellowish and matt. Odourless. Rough touch.	Completely rigid, it breaks when even slight force is applied.

✖: Unacceptable; ✔: Acceptable; ✔✔: Good; ✔✔✔: Excellent.

**Table 2:** Results of f2 processing for drug release from LbL films.

	**ChL/E-a SVF**	**ChL/E-b SVF/SSF**
ChL/E-a SVF/SSF	19.68	59.96
ChL/E-b SVF	73.63	21.21
	**ChT/E-a SVF**	**ChT/E-b SVF/SSF**
ChT/E-a SVF/SSF	14.34	34.43
ChT/E-b SVF	64.99	9.72
	**ChC/E-a SVF**	**ChC/E-b SVF/SSF**
ChC/E-a SVF/SSF	13.00	50.80
ChC/E-b SVF	43.01	11.71

**Table 3:** Results of the cytotoxicity analysis of lactic acid, tartaric acid, citric acid, TEC and ES100 in MT-2, THP-1 and HEC-1A cells. Cytotoxic concentrations 50 (CC_50_) were calculated using GraphPad Prism software.

	CC_50_ µg/mL (CI95%; R^2^)		
	*HEC-1A*	*THP1*	*MT-2*
Lactic acid	>1000	>1000	>1000
Tartaric acid	≈200	>1000	>1000
Citric acid	>1000	≈1000	>1000
TEC	>1000	>1000	≈1000
ES100	>1000	>1000	>1000

**Table 4:** Composition of the chitosan gels.

Batch	Chitosan (%)	Lactic Acid (M)	Tartaric Acid (M)	Citric Acid (M)
gChL-a	3	0.25		
gChL-b	3	0.5		
gChL-c	3	1		
gChT-a	3		0.25	
gChT-b	3		0.5	
gChT-c	3		1	
gChC-a	3			0.25
gChC-b	3			0.5
gChC-c	3			1

**Table 5:** Composition of the chitosan derivative films manufactured with the solvent casting method.

Batch	Chitosan (mg)	Lactic Acid (mg)	Tartaric Acid (mg)	Citric Acid (mg)	TFV (mg)
ChL-a	150	112.5 (0.25 M)			
ChL-b	150	225 (0.5 M)			
ChL-c	150	450 (1 M)			
ChT-a	150		187.5 (0.25 M)		
ChT-b	150		375 (0.5 M)		
ChT-c	150		750 (1 M)		
ChC-a	150			240 (0.25 M)	
ChC-b	150			480 (0.5 M)	
ChC-c	150			960 (1 M)	
ChL-TFV	150	225 (0.5 M)			30
ChT-TFV	150		375 (0.5 M)		30
ChC-TFV	150			480 (0.5 M)	30

**Table 6:** Composition of the LbL films manufactured with the solvent casting method.

		ChL/E-a	ChL/E-b	ChT/E-a	ChT/E-b	ChC/E-a	ChC/E-b
CHITOSAN DERIVATIVE-BASED LAYER	Chitosan (mg)	150	150	150	150	150	150
	Lactic acid (mg)	225	225				
	Tartaric acid (mg)			375	375		
	Citric acid (mg)					480	480
	Tenofovir (mg)	30	30	30	30	30	30
EUDRAGIT^®^ S100-BASED LAYER	Eudragit^®^ S100 (mg)	75	150	75	150	75	150
	Triethylcitrate (mg)	37.5	75	37.5	75	37.5	75
